# MSP is a negative regulator of inflammation and lipogenesis in *ex vivo* models of non-alcoholic steatohepatitis

**DOI:** 10.1038/emm.2016.79

**Published:** 2016-09-09

**Authors:** Dipanjan Chanda, Jieyi Li, Yvonne Oligschlaeger, Mike L J Jeurissen, Tom Houben, Sofie M A Walenbergh, Ronit Shiri-Sverdlov, Dietbert Neumann

**Affiliations:** 1 Department of Molecular Genetics, CARIM School for Cardiovascular Diseases, Maastricht University, Maastricht, The Netherlands; 2 Department of Molecular Genetics, NUTRIM School of Nutrition and Translational Research in Metabolism, Maastricht University, Maastricht, The Netherlands

## Abstract

Non-alcoholic steatohepatitis (NASH), a metabolic disorder consisting of steatosis and inflammation, is considered the hepatic equivalent of metabolic syndrome and can result in irreversible liver damage. Macrophage-stimulating protein (MSP) is a hepatokine that potentially has a beneficial role in hepatic lipid and glucose metabolism via the activation of AMP-activated protein kinase (AMPK). In the current study, we investigated the regulatory role of MSP in the development of inflammation and lipid metabolism in various NASH models, both *in vitro* and *ex vivo*. We observed that MSP treatment activated the AMPK signaling pathway and inhibited lipopolysaccharide (LPS)- and palmitic acid (PA)-induced gene expression of pro-inflammatory cytokines in primary mouse hepatocytes. In addition, MSP treatment resulted in a significant reduction in PA-induced lipid accumulation and inhibited the gene expression of key lipogenic enzymes in HepG2 cells. Upon short hairpin RNA-induced knockdown of RON (the membrane-bound receptor for MSP), the anti-inflammatory and anti-lipogenic effects of MSP were markedly ablated. Finally, to mimic NASH *ex vivo*, we challenged bone marrow-derived macrophages with oxidized low-density lipoprotein (oxLDL) in combination with LPS. OxLDL+LPS exposure led to a marked inhibition of AMPK activity and a robust increase in inflammation. MSP treatment significantly reversed these effects by restoring AMPK activity and by suppressing pro-inflammatory cytokine gene expression and secretion under this condition. Taken together, these data suggest that MSP is an effective inhibitor of inflammation and lipid accumulation in the stressed liver, thereby indicating that MSP has a key regulatory role in NASH.

## Introduction

Non-alcoholic steatohepatitis (NASH) is characterized by excessive hepatic lipid accumulation (steatosis) in addition to inflammation (hepatitis). The transition from steatosis to NASH initiates further severe liver damage and thus represents a crucial step in the pathogenesis of NASH.^1^ Thus far, the triggers for the inflammatory response in the liver are poorly understood. Recent findings suggest that visceral adipose tissue and its secretory products (adipocytokines) are major contributors to inflammation. Increased lipid content in visceral adipose tissue enhances free fatty acid delivery from the adipocytes into the liver, thereby increasing hepatic lipid content and initiating inflammation and insulin resistance.^2^ Recent evidence also indicates that elevated levels of plasma lipopolysaccharide (LPS) secreted from gut microbiota during obesity are a source of liver inflammation.^3^ An increasing number of studies show the involvement of oxidized low-density lipoproteins (oxLDL) in hepatic inflammation, and LPS has been shown to synergize oxLDL uptake in macrophages.^4^ Although the underlying molecular mechanism is currently unclear, oxLDL has emerged as a new risk factor for hepatic inflammation.

Macrophage-stimulating protein (MSP) is constitutively secreted by the liver into the circulating blood as a single chain, biologically inactive pro-MSP. Mature MSP is generated through proteolytic cleavage by trypsin-like serine proteases at extravascular sites and targets macrophages and other cell types. MSP is a ligand for the recepteur d'origine nantais (RON) receptor tyrosine kinase, which is expressed in several tissues, including the liver and brain.^5, 6, 7^ The MSP/RON pair has inhibitory roles in inflammatory responses, such as the production of nitric oxide by stimulated macrophages. Remarkably, MSP-deficient mice on a normal diet develop steatosis. Furthermore, MSP is both necessary and sufficient to induce macrophage polarization into the anti-inflammatory M2 phenotype (as opposed to pro-inflammatory M1 activation), which assists in the attenuation of inflammation.^5, 6^ Previously, we have demonstrated that MSP inhibits gluconeogenesis^8^ and Toll-like receptor signaling^9^ via the activation of AMP-activated protein kinase (AMPK) signaling. AMPK is an important integrator of signals that coordinates energy balance and acts as a protective response to energy stress during metabolic deregulation.^10^ However, an obvious link connecting MSP with inflammation is understudied, and the potential implication of the MSP/RON-AMPK axis in NASH has not been investigated.

In current study, we investigated the role of the MSP/RON-mediated activation of AMPK in the context of inflammation and NASH. We challenged primary mouse hepatocytes, HepG2 cells and bone marrow-derived macrophages (BMDMs) with different triggers for inflammation and NASH. To elucidate the involvement of the MSP signaling pathway under these patho-physiological conditions, we analyzed changes in cell signaling, lipid accumulation and gene expression of inflammatory cytokines and lipogenic enzymes. Our data strongly suggest that MSP, via its receptor RON, activates the downstream AMPK signaling pathway, in turn inhibiting inflammation and excessive lipid accumulation and, thereby, having a crucial role in countering NASH.

## Materials and methods

### Materials

Recombinant human MSP, LPS and oxLDL were purchased from R&D systems. Palmitic acid (PA) was purchased from Sigma-Aldrich (St Louis, MO, USA).

### Cell culture, mice, BMDMs and primary mouse hepatocytes

HepG2 cells (ATCC, Manassas, VA, USA) and age-matched wild-type C57Bl/6 J mice were used for the various experiments. Mice were housed under standard conditions and provided with unlimited access to food and water. Experiments were performed according to Dutch regulations and approved by the Committee for Animal Welfare of Maastricht University. Primary mouse hepatocytes were isolated from C57BL/6 mice using the collagenase perfusion method as previously described.^8^

HepG2 cells and primary mouse hepatocytes were incubated for 24 h with PA (0.5 mM) or for 4 h with LPS (100 ng ml^−1^) in the absence or presence of MSP (100 ng ml^−1^) or 5-aminoimidazole-4-carboxamide ribonucleotide (AICAR) (0.5 mM), as indicated. For treatments involving the AMPK inhibitor Compound C (Comp. C, 10 μM), primary mouse hepatocytes were pretreated with Comp. C for 1 h preceding incubation with PA or LPS in the absence or presence of MSP or AICAR, as indicated in the figures.

BMDMs were isolated from the tibiae and femurs of C57BL/6 mice as previously described.^9, 11^ After attachment, macrophages were incubated with oxLDL (25 μg ml^−1^) for 24 h. Then, the cells were washed and stimulated with LPS in the absence or presence of MSP for 4 h.

Collection of media for ELISA analysis, immunoprecipitation and western blotting, RNA isolation, complementary DNA synthesis and real-time quantitative PCR were performed as previously described.^8, 9, 11^

### Oil Red-O staining

Oil Red-O staining was performed as previously described.^12^ Images acquisition was performed using a bright-field light microscope, and image quantification was performed using the ImageJ program (http://rsbweb.nih.gov/ij/).

### Lentiviral infection

pLKO.1 puro was a gift from Bob Weinberg (Addgene plasmid # 8453). HEK-293T cells were co-transfected with psPAX2 and pMD2.G lentivirus packaging vectors with pLKO.1 scramble (shScr) or pLKO.1 RONα (shRONα) using Lipofectamine 2000 (Invitrogen, Carlsbad, CA, USA) according to the manufacturer's protocol. After 48 h infection, the lentivirus particles were collected from the HEK-293T cells, and HepG2 cells were infected with these particles. Infected cells were selected for puromycin resistance (4 μg ml^−1^) for 5 days, and western blotting analysis was performed to determine knockdown efficiency.

### Statistical analysis

The data were analyzed using Graphpad Prism 4.0.3 (GraphPad Software, Inc., La Jolla, CA, USA). Unpaired Student's *t*-tests were performed, and the data are expressed as the mean±s.e.m. The level of significance was set at *P<*0.05.

## Results

### MSP inhibits PA- and LPS-induced inflammation via the activation of the AMPK signaling pathway

**Figure 1:**
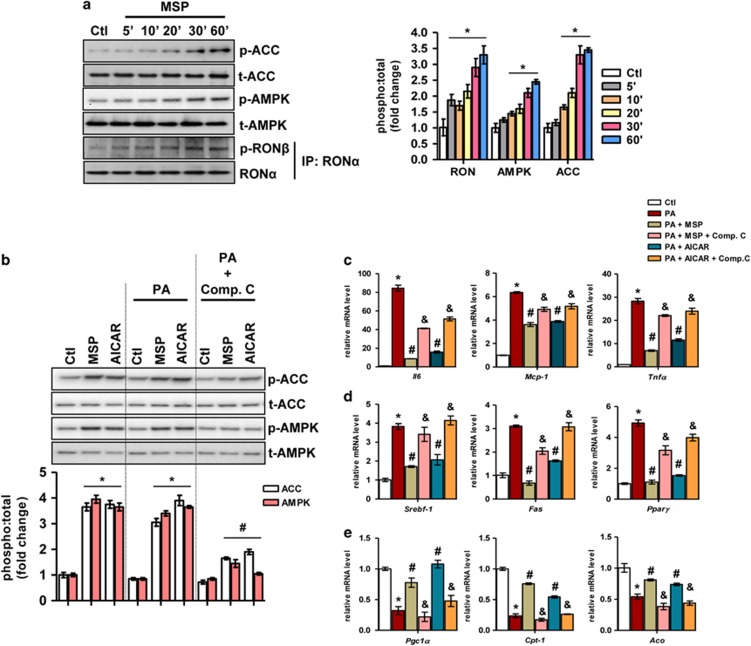
MSP alleviates PA-induced inflammation in primary mouse hepatocytes. (**a**) Representative western blotting analysis (left) and quantification of the fold change relative to the vehicle control (right) of RON, AMPK and ACC phosphorylation upon MSP treatment for the indicated amount of time. Data are expressed as the mean±s.e.m. (*n*=6). *P<0.05 vs Ctl. (**b**) Representative western blot (top) and quantification of the fold change relative to the vehicle control (bottom) of AMPK and ACC phosphorylation for the indicated treatments. Data are expressed as the mean±s.e.m. (*n*=6). *P<0.05 vs Ctl; ^#^*P<*0.05 vs PA. (**c–e**) Real-time quantitative PCR analysis of genes involved in inflammation (**c**), lipogenesis (**d**) and fatty acid oxidation (**e**) from the hepatocytes treated as indicated. Each value indicates the amount of mRNA relative to the vehicle control-treated hepatocytes. *Cyclophilin A* was used as the invariant control. Data are expressed as the mean±s.e.m. (*n*=6). *P<0.01 vs Ctl; ^#^*P<*0.05 vs PA; ^&^*P<*0.05 vs PA±MSP or PA±AICAR. ACC, acetyl CoA carboxylase; AMPK, AMP-activated protein kinase; Ctl, control; MSP, macrophage-stimulating protein; PA, palmitic acid; RON, recepteur d'origine nantais.

**Figure 2:**
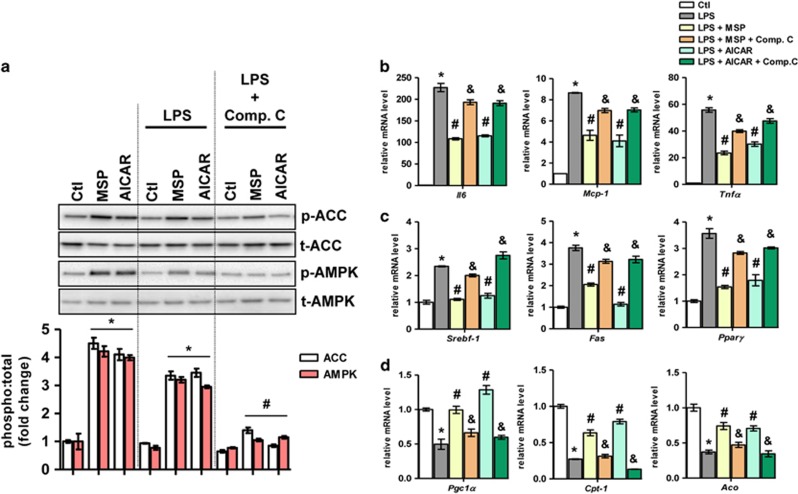
MSP ameliorates LPS-induced inflammation in primary mouse hepatocytes. (**a**) Representative western blot (top) and quantification of the fold change relative to the vehicle control (bottom) of AMPK and ACC phosphorylation for the indicated treatments. Data are expressed as the mean±s.e.m. (*n*=6). *P<0.05 vs Ctl; ^#^*P<*0.05 vs LPS. (**b**–**d**) Real-time qPCR analysis of genes involved in inflammation (**b**), lipogenesis (**c**), and fatty acid oxidation (**d**) from the hepatocytes treated as indicated. Each value indicates the amount of mRNA relative to the vehicle control-treated hepatocytes. *Cyclophilin A* was used as the invariant control. Data are expressed as the mean±s.e.m. (*n*=6). *P<0.01 vs Ctl; ^#^*P<*0.05 vs LPS; ^&^*P<*0.05 vs LPS±MSP or LPS±AICAR. ACC, acetyl CoA carboxylase; AMPK, AMP-activated protein kinase; Ctl, control; LPS, lipopolysaccharide; MSP, macrophage-stimulating protein; qPCR, quantitative PCR.

Previously, we demonstrated that MSP activates AMPK signaling in the human hepatoma cell line HepG2 and in primary rat hepatocytes.^8^ In primary mouse hepatocytes, we confirmed that 5–60 min incubations with MSP (100 ng ml^−1^) enhanced the phosphorylation of AMPK and of the downstream AMPK target acetyl CoA carboxylase (ACC) via the phosphorylation and activation of the RON receptor (Figure 1a). Previous studies have demonstrated that both PA (the predominant free fatty acid in circulation) and LPS induce an inflammatory response in hepatocytes.^3, 13^ Conversely, AMPK activation is considered to have potential therapeutic benefits owing to the anti-inflammatory properties of AMPK.^9, 14, 15, 16^ Thus, primary hepatocytes were challenged with PA or LPS in the absence or the presence of MSP or a well-established AMPK activator, AICAR (Figures 1b–e and 2). In addition, to reconfirm MSP-induced activation of the AMPK signaling pathway, hepatocytes were pretreated with the AMPK inhibitor Comp. C in the presence of MSP and AICAR. Both MSP and AICAR increased the phosphorylation levels of AMPK and ACC in PA- and LPS-challenged hepatocytes (Figures 1b and 2a, respectively). However, this effect was significantly attenuated upon pretreatment with Comp. C. PA- and LPS-challenged hepatocytes (Figures 1c and 2b, respectively) demonstrated a marked increase in the gene expression of key pro-inflammatory markers, tumor necrosis factor-α (*Tnfα*), interleukin-6 (*Il6*) and monocyte chemoattractant protein-1 (*Mcp-1*). This increase in pro-inflammatory marker gene expression was significantly ablated in the presence of either MSP or AICAR under these conditions. However, consistent with the inhibition of AMPK and ACC phosphorylation, pretreatment with Comp. C markedly reversed the inhibitory effect of either MSP or AICAR on *Tnfα, Il6* and *Mcp-1* gene expression levels.

Because it has been well established that peripheral AMPK activation can promote fatty acid oxidation by phosphorylating and inactivating ACC as well as inhibiting fatty acid synthesis,^14, 15, 16^ we determined whether MSP-induced AMPK phosphorylation in these inflammation-challenged hepatocytes can affect the expression of genes involved in lipid metabolism. To test this hypothesis, we investigated the expression of lipogenic genes in PA- and LPS-challenged hepatocytes treated with MSP or AICAR in the absence or the presence of AMPK inhibition. As demonstrated, PA- and LPS-challenged hepatocytes (Figures 1d and 2c, respectively) demonstrated a marked increase in the expression of key lipogenic genes—sterol regulatory element-binding factor 1 (*Srebf-1*), fatty acid synthase (*Fas*) and peroxisome proliferator-activated receptor-α (*Ppar-α*). MSP (or AICAR) treatment resulted in a marked reduction of *Srebf-1*, *Fas* and *Ppar-α* expression levels, and this anti-lipogenic effect of MSP (or AICAR) was significantly attenuated upon AMPK inhibition by Comp. C. In contrast, PA- and LPS-induced inhibition (Figures 1e and 2d, respectively) of the expression of key fatty acid oxidation genes, such as acyl-CoA oxidase (*Aco*), carnitine palmitoyltransferase I (*Cpt-1*) and peroxisome proliferator-activated receptor-γ (PPARγ) coactivator-1α (*Pgc-1α*), was markedly reversed with MSP or AICAR treatment. Consistent with previous observations, AMPK inhibition following pretreatment with Comp. C under these conditions reversed the stimulation of fatty acid oxidation observed with either MSP or AICAR treatment. Taken together, these findings suggest that MSP ameliorated *ex vivo* NASH-mimicking conditions by enhancing fatty acid oxidation and by repressing lipogenesis and the inflammatory response via the activation of the AMPK signaling pathway.

### MSP inhibits PA-induced lipid accumulation

**Figure 3:**
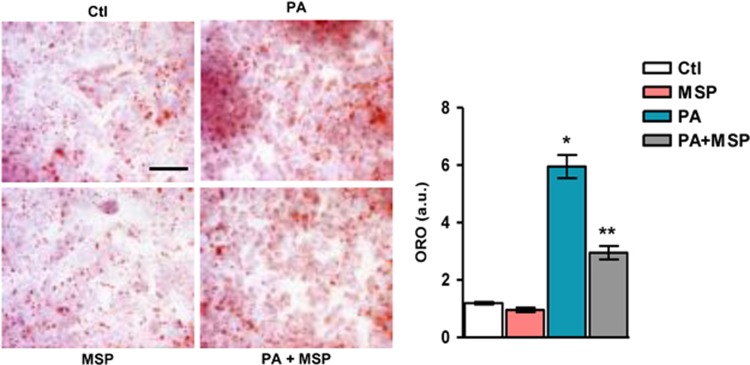
MSP treatment reduces neutral lipid accumulation in HepG2 cells. Visualization (representative images) and quantification of neutral lipids by ORO analysis in HepG2 cells challenged with PA, in the absence or presence of MSP. Scale bars, 100 μm; × 40 magnification; a.u., arbitrary units. Data are expressed as the mean±s.e.m. of three independent experiments. *P<0.01 vs Ctl; **P<0.01 vs PA. Ctl, control; MSP, macrophage-stimulating protein; ORO, Oil Red-O; PA, palmitic acid.

Recent studies have demonstrated that an aberrant increase in lipid accumulation is a characteristic of metabolic diseases, such as type 2 diabetes, NASH and metabolic syndrome.^2, 12, 13^ Thus, using Oil Red-O staining for lipid droplets, we examined the effect of MSP co-treatment on PA-induced lipid accumulation. PA exposure led to a marked increase in lipid droplets in these cells (~sixfold compared with the control), suggesting a robust increase in lipid accumulation (Figure 3). However, co-treatment with MSP significantly reduced lipid accumulation (~3.5-fold compared with PA-challenged cells), thereby implying that MSP inhibits excessive lipid accumulation, potentially via the enhancement of lipid oxidation and/or inhibition of lipogenesis to counter inflammation.

### Anti-inflammatory and anti-lipogenic effect of MSP is reversed upon knockdown of the RON receptor

**Figure 4:**
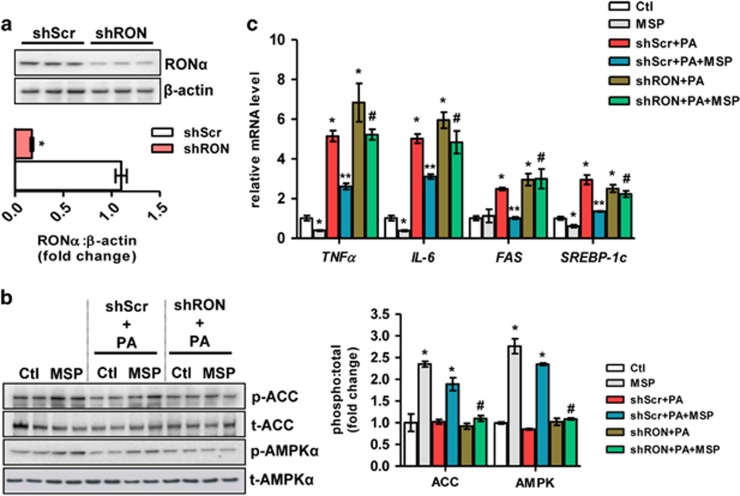
RON receptor relays the downstream effects of MSP in HepG2 cells. (**a**) Representative western blot of shRNA-induced knockdown of RON (top) and quantification of the fold change relative to shScr (bottom). Data are expressed as the mean±s.e.m. of three independent experiments. *P<0.01 vs shScr. (**b**) Representative western blot (left) and quantification of the fold change relative to Ctl (bottom) of AMPK and ACC phosphorylation in cells challenged with PA, in the absence or presence of MSP, in RON knockdown cells. Data are expressed as the mean±s.e.m. of three independent experiments. *P<0.01 vs Ctl; ^#^*P<*0.05 vs shScr±PA±MSP. (**c**) Real-time qPCR analysis of genes involved in inflammation (*TNFα, IL-6*) and lipogenesis (*FAS*, *SREBP-1c*) in cells challenged with PA, in the absence or presence of MSP, in RON knockdown cells. Values are expressed as the mean±s.e.m. of three independent experiments. *P<0.01 vs Ctl; **P<0.05 vs shScr+PA; ^#^*P<*0.05 vs shScr+PA+MSP. ACC, acetyl CoA carboxylase; AMPK, AMP-activated protein kinase; Ctl, control; MSP, macrophage-stimulating protein; PA, palmitic acid; qPCR, quantitative PCR; RON, recepteur d'origine nantais.

Next, to determine whether MSP activates AMPK via its membrane-bound RON receptor and whether MSP exerts anti-inflammatory and anti-lipogenic effects, we employed lentivirus-mediated knockdown of the RON receptor (shRON) in HepG2 cells, which markedly reduced RON receptor levels (RONα) compared with the scrambled, non-specific virus-infected cells (shScr) (Figure 4a). As expected, in shScr-infected cells, MSP treatment led to a significant increase in AMPK and ACC phosphorylation in the absence or presence of PA. However, the activation of the AMPK signaling pathway was significantly diminished in shRON-infected cells, confirming that MSP exerts its effect via its RON receptor (Figure 4b). In the presence of shScr, MSP co-treatment resulted in a significant reduction in the PA-induced gene expression of pro-inflammatory markers *TNFα* and *IL-6*, along with the key lipogenic enzymes *SREBP-1c* and *FAS* (Figure 4c). However, in shRON-infected cells, the anti-lipogenic and anti-inflammatory effect of MSP co-treatment is dramatically abolished, thus confirming that MSP regulates lipogenesis and inflammation via its RON receptor.

### MSP inhibits inflammation in an *ex vivo* model mimicking NASH

**Figure 5:**
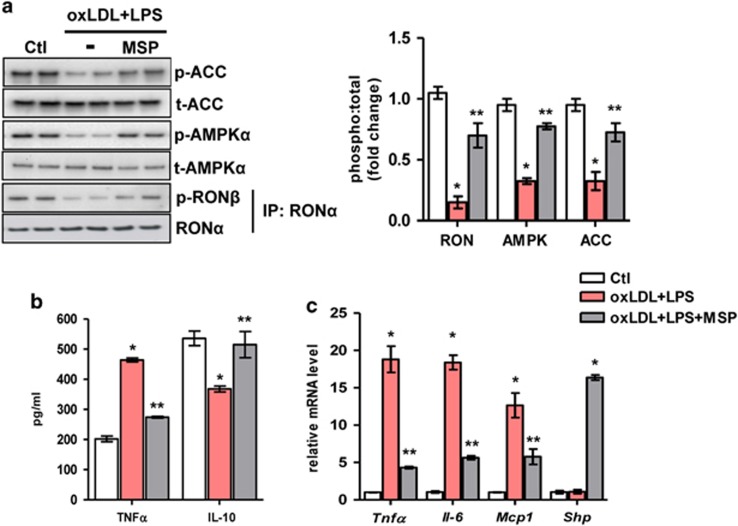
MSP exerts anti-inflammatory effects in BMDMs. (**a**) Representative western blot (left) and quantification of the fold change relative to vehicle control (right) of RON, AMPK and ACC phosphorylation (**b**) ELISA analysis of TNFα and IL-10 production, and (**c**) Gene expression of pro-inflammatory cytokines *Tnfα*, *Il-6*, *Mcp-1* and orphan nuclear receptor *Shp* in oxLDL+LPS challenged BMDMs, in the absence or presence of MSP. Values are expressed as the mean±s.e.m., *n*=5 per group. *P<0.01 vs Ctl; **P<0.05 vs oxLDL+LPS. ACC, acetyl CoA carboxylase; AMPK, AMP-activated protein kinase; BMDM, bone marrow-derived macrophage; Ctl, control; IL-10, interleukin 10; LPS, lipopolysaccharide; *Mcp-1*, monocyte chemoattractant protein-1; MSP, macrophage-stimulating protein; oxLDL, oxidized low-density lipoprotein; RON, recepteur d'origine nantais; TNFα, tumor necrosis factor-α.

Macrophages have a pivotal role in hepatic inflammation and in the subsequent development of NASH. Recent data show the involvement and contribution of oxLDL in hepatic inflammation, thus implicating it to be a new risk factor for hepatic inflammation.^1^ In addition, it has been shown that LPS augments the uptake of oxLDL in macrophages.^4^ Therefore, we examined the effect of MSP on oxLDL+LPS-induced inflammation in BMDMs. BMDMs were treated with oxLDL for 24 h followed by LPS treatment for 4 h in the absence or presence of MSP. RON receptor, AMPK and ACC phosphorylation was significantly inhibited by oxLDL+LPS exposure, and upon MSP co-treatment, this inhibitory effect on the activation of RON and the downstream activation of the AMPK signaling pathway was markedly reversed (Figure 5a). Next, we observed that oxLDL+LPS exposure led to a significant increase in TNFα production and decreased the production of the anti-inflammatory cytokine interleukin 10 (IL-10). MSP co-treatment under this condition resulted in a marked reversal of TNFα production and concomitantly induced the production of IL-10 (Figure 5b), indicating an anti-inflammatory role of MSP. Finally, gene expression analysis demonstrated that MSP co-treatment resulted in a significant reduction in oxLDL+LPS-induced *Tnfα*, *Il-6* and *Mcp-1* mRNA levels in BMDMs (Figure 5c). Moreover, as expected from our previous findings,^9^ MSP treatments led to a strong induction of orphan nuclear receptor small heterodimer partner (*Shp*; Nr0b2) gene expression. Overall, these results suggest that MSP reduces inflammation to alleviate NASH, both *in vitro* and *ex vivo*.

## Discussion

**Figure 6:**
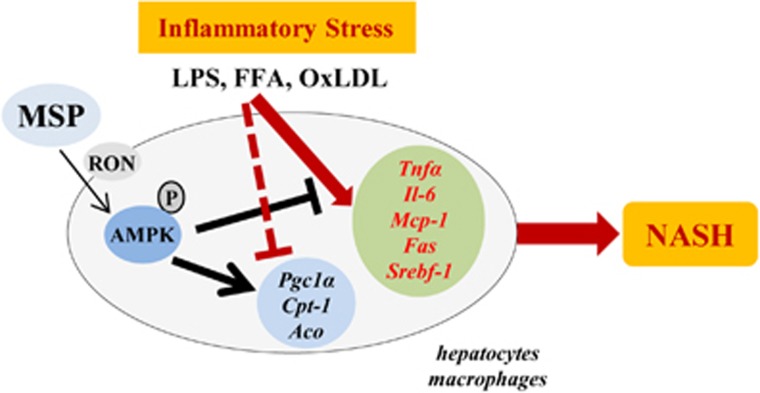
Schematic model representing MSP as a negative regulator of NASH in hepatocytes and macrophages. Mediators of inflammatory stress—FFA, LPS or oxLDL—induce gene expression of key pro-inflammatory (*Tnfα*, *Il-6* and *Mcp-1*) and lipogenic (*Srebf-1* and *Fas*) markers in hepatocytes and macrophages, precipitating a pathophysiological condition termed NASH. MSP, a hepatokine and ligand for the RON receptor tyrosine kinase, activates AMPK signaling pathway, in turn stimulating fatty acid oxidation (via the upregulation of *Pgc-1α*, *Cpt-1* and *Aco*) and inhibiting inflammation and lipogenesis in *ex vivo* and *in vitro* models mimicking NASH. AMPK, AMP-activated protein kinase; *Aco*, acyl-CoA oxidase; *Cpt-1*, carnitine palmitoyltransferase I; FFA, free fatty acid; IL-6, interleukin 6; LPS, lipopolysaccharide; *Mcp-1*, monocyte chemoattractant protein-1; MSP, macrophage-stimulating protein; oxLDL, oxidized low-density lipoprotein; *Pgc-1α*, peroxisome proliferator-activated receptor-γ (PPARγ) coactivator-1α RON, recepteur d'origine nantais; TNFα, tumor necrosis factor-α.

NASH is a feature of the metabolic syndrome, and as such, it is strongly associated with insulin resistance. Hepatic steatosis is characterized by a higher dietary fat intake, increased *de novo* lipogenesis, and increased lipolysis in adipose tissue, leading to an imbalance between lipid storage and lipid removal. Furthermore, macrophages and other immune cells are recruited to the liver and secrete pro-inflammatory cytokines, perpetuating chronic hepatic inflammation and eventually progressing toward cirrhosis and hepatocellular carcinoma.^1, 2, 3^ Currently, there is no effective treatment for NASH. In current study, we demonstrated for the first time that MSP acts as a key negative regulator of inflammation and lipogenesis by activating the AMPK signaling pathway in hepatocytes and macrophages. Our data indicate that MSP has anti-inflammatory and anti-lipogenic properties and stimulates fatty acid oxidation under metabolic challenge conditions, suggesting that MSP has a beneficial role in countering NASH (Figure 6).

Previously, we demonstrated that MSP activates the AMPK signaling pathway in primary rat hepatocytes and various hepatic cell lines.^8, 9^ As a reconfirmation of this phenomenon, we observed a similar effect of MSP on AMPK activation in primary mouse hepatocytes, which is comparable to the well-recognized AMPK activator AICAR. Several previous reports have indicated that MSP has a key role in regulating inflammation,^7, 9, 17, 18^ particularly during LPS-induced endotoxemia. However, very little is known regarding the regulatory role of MSP in NASH. To address this question, we challenged primary hepatocytes and human hepatoma HepG2 cells, in addition to LPS exposure, with PA. PA is the predominant-free fatty acid in circulation that mimics the diet-induced obesity models *in vivo* and is an established model to study hepatic inflammation, both *in vitro* and *ex vivo*.^19^ Our results demonstrated that MSP countered and reversed the pro-inflammatory and lipogenic effects of both PA and LPS. However, MSP reversed the inhibition of fatty acid oxidation upon PA and LPS exposure. Consistent with previous findings implicating that AMPK, which is a key metabolic regulator, has anti-inflammatory effects,^9, 14, 15, 16^ we observed that activation of AMPK by MSP precedes its anti-inflammatory and anti-lipogenic effects under these conditions. Thus, our findings suggest that the MSP-induced activation of AMPK initiates a crucial chain of signaling to reverse hepatic inflammation.

An aberrant increase in lipogenesis and lipid accumulation in hepatocytes is a hallmark of metabolic syndrome, including NASH.^2, 12, 13^ It has been shown that MSP^−/−^ mice develop hepatic steatosis with an accumulation of lipid droplets in hepatocytes under normal chow diet,^20^ indicating a key role of MSP in regulating hepatic lipid metabolism. However, to the best of our knowledge, the anti-inflammatory aspect of MSP, in the context of NASH, has never been addressed. Our results from neutral lipid staining demonstrated that MSP treatment resulted in a significant reduction in PA-induced lipid accumulation in HepG2 cells. Furthermore, we observed that MSP treatment inhibits PA-induced hepatic lipogenesis via downregulating the gene expression of key lipogenic enzymes, *SREBP-1c* and *FAS*. Because AMPK is known to inhibit lipogenesis by phosphorylating and downregulating SREBP-1c,^21^ we anticipated that this anti-lipogenic effect of MSP occurs via the activation of AMPK. Indeed, knockdown of RON, the membrane-bound receptor for MSP, led to a marked decrease in the MSP-induced activation of AMPK and significantly reversed the anti-inflammatory and anti-lipogenic effects of MSP in PA-challenged cells. Conversely, a recent study using a high-fat diet model demonstrated that *Ron*^−*/*−^ mice are protected against obesity and steatosis.^22^ However, the same authors have previously shown that a Ron receptor deficiency results in the potentiation of the inflammatory response and increased mortality resulting from LPS-induced endotoxemia, ^18^ which supports our current findings. Overall, these results indicated that the MSP-AMPK axis has a key role in regulating hepatic lipid metabolism and inflammation in metabolically challenging conditions.

Recent findings based on molecular and clinical approaches suggest that oxLDL has a critical role in the development of hepatic steatosis and inflammation in macrophages, and the inhibition of the oxLDL effect is expected to be beneficial in NASH.^23, 24^ Moreover, it has been shown that LPS synergizes oxLDL uptake in macrophages to exacerbate inflammation.^4^ Previously, we have demonstrated that MSP, via AMPK activation, induces orphan nuclear receptor Shp to inhibit sepsis-induced inflammation in BMDMs, and this effect of MSP was ablated in Shp ^−/−^ mice.^9^ Here, we demonstrate that MSP strongly counteracts oxLDL+LPS-induced inflammation in BMDMs by activating the AMPK signaling pathway. In addition, MSP inhibits the production of the pro-inflammatory cytokine TNFα and induces the production of the anti-inflammatory IL-10 in this condition. These findings are supported by previous reports indicating that RON receptor signaling inhibits macrophage-dependent pro-inflammatory cytokine production during endotoxemia.^7, 9^ Thus, our results indicate that the MSP–AMPK axis exhibits beneficial anti-inflammatory properties and protects macrophages during inflammatory stress conditions.

Taken together, our current findings unraveled a key role for MSP in the context of inflammation and steatosis. Here, we provide novel insight into the role of MSP in regulating lipid metabolism, and using various metabolic challenge models of inflammation, we elucidated the molecular mechanism of MSP action and its beneficial anti-inflammatory effect in NASH, thereby indicating that MSP can be a promising therapeutic option for NASH.
